# Editorial: Advances in volume electron microscopy for brain imaging: methods, applications, and affordability

**DOI:** 10.3389/fnins.2025.1561852

**Published:** 2025-02-05

**Authors:** Corrado Calì, Xueying Wang

**Affiliations:** ^1^Department of Neuroscience “Rita Levi Montalcini”, University of Turin, Turin, Italy; ^2^Neuroscience Institute Cavalieri, Orbassano, Italy; ^3^Moderna, Inc., Cambridge, MA, United States

**Keywords:** volume electron microscopy (vEM), resin, gliotransmission, image registration, 3D reconstruction, ADHD, embedding, synaptic plasticity

## Introduction

Volume electron microscopy (vEM) has emerged as a cornerstone of modern neuroscience, providing insights into the brain's ultrastructure with nanometer precision. The combination of high spatial resolution and volumetric imaging has enabled detailed reconstructions of neural circuits, cellular morphology, and synaptic architecture, offering a deeper understanding of the structure-function relationship in the brain (Denk and Horstmann, 2004; Knott and Genoud, 2013). This Research Topic of Frontiers in Neuroscience showcases recent advancements in vEM, emphasizing the field's capacity to address both methodological challenges and biological questions.

The contributions in this Research Topic reflect two primary themes: (1) advancements in vEM methodologies, including computational and material innovations, and (2) biological insights enabled by vEM, demonstrating its utility in understanding neurodevelopment, glial function, and therapeutic interventions.

## Advancing methodological capabilities

The technical demands of vEM, particularly for data alignment and sample preparation, remain a bottleneck for many laboratories. Innovations in these areas are essential for broadening the accessibility and impact of vEM.

Watkins et al. introduce msemalign, a computational pipeline for aligning petabyte-scale datasets generated by serial section multibeam scanning electron microscopy (ssmSEM). This tool addresses the challenges of processing massive image datasets, emphasizing scalability and ease of use. Unlike existing database-driven solutions, msemalign minimizes computational overhead, making it more accessible to labs with limited resources. By aligning datasets with minimal distortion and maintaining continuity of tissue structures, this pipeline facilitates large-scale neural circuit reconstruction.

Tegethoff and Briggman focus on embedding resins, a critical yet underexplored aspect of vEM sample preparation. Their study quantifies resin hardness, cutting forces, and curing uniformity, providing researchers with practical metrics for selecting optimal resins. Consistent ultrathin sectioning is crucial for achieving high-resolution volumetric imaging, and this work sets a new benchmark for reproducibility in vEM datasets.

## Biological insights enabled by vEM

Beyond technical advancements, vEM continues to illuminate fundamental biological processes. In this Research Topic, Calì revisits the debate on astrocytic gliotransmission, presenting ultrastructural evidence for synaptic-like microvesicles (SLMVs) in astrocytes. The potential role of regulated exocytosis in modulating synaptic activity has been a contentious topic (Bezzi et al., 2004; Savtchouk and Volterra, 2018). By leveraging vEM to visualize nanometer-scale organelles in astrocytic processes, this study provides fresh insights into the cellular mechanisms underpinning neuroglia communication.

Complementing this, Sun et al. explore the therapeutic potential of Rehmanniae Radix Preparata (RRP), a traditional Chinese medicine, in a rat model of ADHD. Combining behavioral analyses, molecular assays, and vEM imaging, they demonstrate that RRP improves hippocampal neurodevelopment, synaptic plasticity, and behavioral outcomes. These findings highlight the versatility of vEM in integrating structural and functional insights, advancing both basic neuroscience and translational research (Plessen et al., 2006; Hoogman et al., 2019).

## The future of vEM

The contributions in this Research Topic underscore the transformative potential of vEM. By addressing methodological challenges, such as data processing and sample preparation, researchers are making vEM more accessible and scalable. Moreover, the integration of vEM with other modalities, including light microscopy and molecular profiling, is creating new opportunities for multi-scale analyses of brain structure and function (Oh et al., 2014; Kaynig et al., 2015).

As the field progresses, affordability and accessibility remain critical challenges. The development of open-source tools, standardized protocols, and cost-effective imaging platforms will be essential for democratizing vEM. With continued innovation and collaboration, vEM is poised to unlock new frontiers in neuroscience, offering unprecedented insights into the brain's complexity.
